# Seroprevalence of Peste des Petits Ruminants Virus Antibodies and Associated Factors in Small Ruminants Sampled Around Protected Wildlife Areas in Pakistan

**DOI:** 10.3390/vetsci13101105

**Published:** 2026-10-16

**Authors:** Abdul Kabir, Muhammad Abubakar, Asghar Ali Kamboh, Shumaila Manzoor, Muhammad Farooq, Abdulkareem Mohammed Matar

**Affiliations:** 1 Department of Veterinary Microbiology, Faculty of Animal Husbandry and Veterinary Sciences, Sindh Agriculture University, Tandojam 70060, Pakistan; 2 National Veterinary Laboratory, Park Road, Islamabad 44000, Pakistan; 3 Health Research Innovation Center, Faculty of Veterinary Medicine, University of Calgary, Calgary, AB T2N 4N1, Canada; 4 Department of Animal Production, College of Food and Agriculture Sciences, King Saud University, P.O. Box 2460, Riyadh 11451, Saudi Arabia

**Keywords:** Peste des Petits Ruminants, small ruminants, livestock–wildlife interface, seroprevalence

## Abstract

Peste des Petits Ruminants (PPR) threatens small-ruminant production in Pakistan, particularly where livestock grazing overlaps with wildlife habitats. In this exploratory cross-sectional study (January–June 2024), 1939 sera from goats (*n* = 1327) and sheep (*n* = 612) in 56 herds, purposively selected near eight protected wildlife areas, were tested for antibodies against PPR virus (PPRV) using a competitive ELISA. Vaccinated and unvaccinated animals were included, and the assay cannot distinguish vaccine-induced from infection-induced antibodies. The overall apparent antibody seroprevalence was 38.5% (747/1939; 95% CI: 36.4–40.7%) (naive 95% CI; it ignores clustering and understates uncertainty, and between-site heterogeneity was very high, I^2^ = 94%), ranging from 16.7% in Peshawar to 73.0% at Kirthar National Park, Sindh. Crude odds of seropositivity were higher in goats than in sheep (44.8% vs. 25.0%; OR = 2.43, 95% CI: 1.97–3.01), in animals older than three years than in those aged > 1–3 years (52.0% vs. 33.8%; OR = 2.13, 95% CI: 1.69–2.67), and in herds with more than 100 animals than in herds with 50–100 animals (43.3% vs. 35.7%; OR = 1.38, 95% CI: 1.11–1.72). After adjustment for site, goats remained more likely to be seropositive than sheep (OR = 2.19, 95% CI: 1.76–2.74), although the species difference varied between sites (heterogeneity *p* = 0.005). Herd-level clustering could not be modelled; associations with herd-level factors (herd size, vaccination status) were sensitive to plausible within-herd correlation and are exploratory. Vaccination categories are reported descriptively only. PPRV antibodies were common in small ruminants sampled near the selected protected areas. However, wildlife were not sampled, distance categories were confounded with site, and results are not nationally representative; transmission at the livestock–wildlife interface was not demonstrated. Targeted surveillance and integrated livestock–wildlife studies are warranted.

## Simple Summary

Peste des Petits Ruminants is a serious viral disease of sheep and goats that causes heavy losses for farming families. Where grazing lands border protected wildlife areas, the disease may also threaten wild animals. We collected blood from 1939 sheep and goats on 56 farms near eight protected wildlife areas in Pakistan during the first half of 2024 and tested it for antibodies against the virus. About four in ten animals (38.5%) had antibodies, which may result from past infection or from vaccination. Antibodies were more common in goats than in sheep, in animals older than three years and in larger herds, and they differed widely between sites. We did not test wild animals, and farms were chosen on purpose rather than at random, so the results do not show that the disease spreads between wildlife and livestock and do not represent the whole country. The findings point to areas and animal groups where closer monitoring is needed, and they show why studies that include both livestock and wildlife are required to support disease control.

## 1. Introduction

Peste des Petits Ruminants (PPR) is a highly contagious viral disease of small ruminants caused by the Peste des Petits Ruminants Virus, a member of the genus *Morbillivirus* in the family *Paramyxoviridae* [1]. The disease primarily affects goats and sheep, although several wild ungulates such as ibex and antelopes can also be infected, often resulting in high morbidity and mortality [2]. Initially confined to Africa and the Middle East, PPR has now spread widely across Asia, including Pakistan, where it is considered endemic [3].

PPR causes substantial economic losses due to high mortality, reduced milk and meat production, increased treatment costs, and restrictions on livestock trade [4,5]. In Pakistan, small ruminants play a vital role in rural livelihoods and are often referred to as “living banks” because of their contribution to household income and food security [6]. Consequently, outbreaks of PPR can have serious impacts on the national livestock sector and smallholder farmers [7].

Transmission of PPR mainly occurs through direct contact with infected animals via respiratory secretions, while indirect transmission may occur through contaminated feed, water, and equipment [8]. High livestock density, seasonal migration, and cross-border animal movement further complicate disease control. Moreover, spillovers of infection from domestic animals to wild ungulates, including ibex and wild goats, have been reported in several regions, posing potential risks to both livestock production and wildlife conservation [9].

Although an effective vaccine is available, control of PPR in Pakistan remains challenging due to limited veterinary infrastructure, inadequate vaccination coverage, and low awareness among livestock farmers [10]. The global eradication strategy led by World Organization for Animal Health and Food and Agriculture Organization aims to eliminate PPR worldwide by 2030 through vaccination, surveillance, and improved biosecurity measures.

Despite several studies reporting the prevalence of PPR in different regions of Pakistan, limited information is available regarding its occurrence and associated factors at the livestock–wildlife interface, particularly around wildlife parks where domestic and wild ruminants may share grazing areas and water sources [11]. Such interfaces may serve as potential hotspots for cross-species transmission but remain poorly investigated.

Therefore, the present study aimed to estimate the apparent seroprevalence of PPRV antibodies and identify factors associated with seropositivity among small ruminants reared around wildlife parks in Pakistan. The findings may contribute to improved surveillance and targeted vaccination strategies for effective disease control in both livestock and wildlife populations.

## 2. Materials and Methods

### 2.1. Study Area

**Figure 1:**
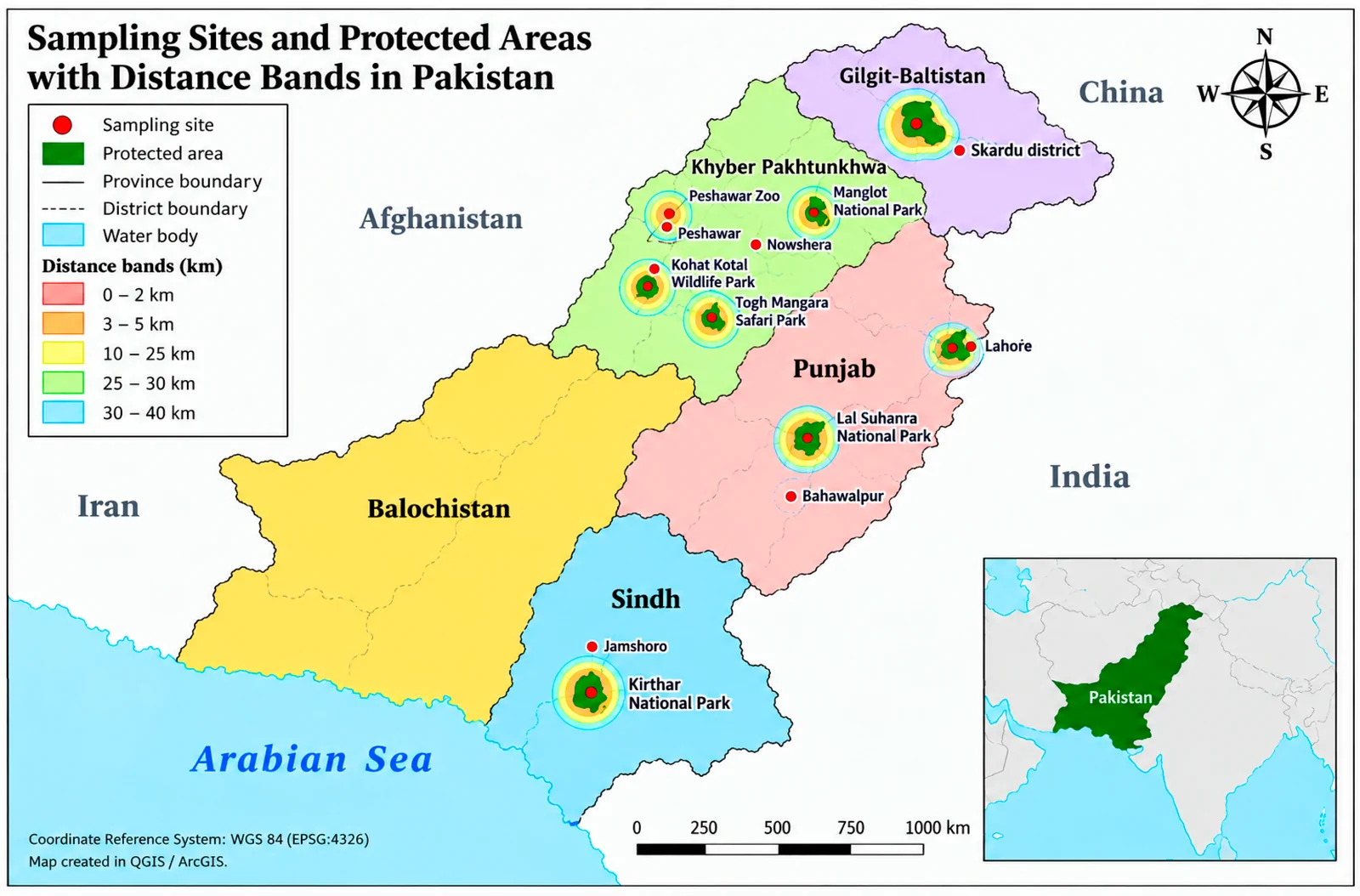
Sampling sites and protected areas in Pakistan. Coordinates represent the approximate centroid of the primary grazing area at each sampling site, recorded by handheld GPS. The distance category for each site was determined as the straight-line distance from that grazing area to the nearest boundary of the protected area, measured using Google Earth.

The study was conducted in three provinces of Pakistan and the administrative territory of Gilgit-Baltistan, representing distinct agro-ecological zones: Punjab (Lahore and Bahawalpur districts), Sindh (Jamshoro district, in the vicinity of Kirthar National Park), Khyber Pakhtunkhwa (Peshawar, Nowshera, and Kohat districts), and Gilgit-Baltistan (Skardu district). Sampling was performed in areas in the vicinity of major protected wildlife areas, including Lal Suhanra National Park, Kirthar National Park, Manglot National Park, Kotal Wildlife Park, Togh Mangara Safari Park and Game Reserve, Lahore Safari/Wildlife Park, Peshawar Zoo, and Deosai National Park. These sites were selected purposively to represent (i) different agro-ecological zones, (ii) varying livestock production systems, and (iii) differing degrees of proximity to protected wildlife habitats. The geographical distribution of the study sites is shown in Figure 1.

### 2.2. Study Animals and Eligibility Criteria

The study population consisted of sheep and goats raised under extensive or semi-intensive production systems in areas located within the predefined distance categories from the selected protected wildlife areas (0–2, 3–5, 10–25, 25–30, and 30–40 km). Breeds represented included Damani, Dera Din Panah (DDP), Kamori, Tapri, Beetal, Teddy, Baltistani, Gojal, Balkhi, Kaghani, Michni, Thalli, Kachhi, Lohi, and Kajli.

Animals were eligible for inclusion if they were apparently healthy at the time of sampling, aged ≥ 3 months, and belonged to households that had resided in the study area for at least 12 months. Animals that did not meet these criteria or for which the owner did not consent to sampling were excluded. A total of 56 herds and 1939 animals (1327 goats and 612 sheep) were enrolled.

Age was recorded from owner recall and, where available, dentition, and was categorized as ≤1 year, >1–3 years, and >3 years for analysis. Sex and reproductive status (for females) were recorded for each animal

### 2.3. Study Design and Sampling Strategy

A cross-sectional study was conducted from January to June 2024. A multistage sampling strategy was applied:

Stage 1 (region and site selection): Four administrative units (three provinces and Gilgit-Baltistan) and eight sampling sites were selected purposively to represent distinct agro-ecological zones and varying degrees of proximity to protected wildlife areas. Purposive selection was used because the study specifically targeted livestock–wildlife interface settings rather than aiming to generate a nationally representative estimate.

Stage 2 (herd selection): Within each district, eligible herds were identified and enumerated with the assistance of local veterinary officers. Herds were selected purposively on the basis of (i) owner willingness to participate, (ii) accessibility of the herd to the field team, (iii) proximity to a protected wildlife area, and (iv) herd size sufficient to permit sampling of multiple animals. Herds of all size categories were included so that herd size could be examined as a factor associated with antibody seropositivity. Stage 3 (animal selection): Within each selected herd, every available animal meeting the eligibility criteria was included in the study. Between 24 and 41 animals were sampled per herd (overall mean ~35), depending on herd size and the number of animals available on the day of sampling. In herds containing more than 100 animals, a maximum of approximately 40 animals per herd was sampled, selected by systematic sampling across the herd in the order in which animals were presented; all other herds contributed all eligible animals present on the day of sampling. A total of 56 herds and 1939 animals were enrolled. Because the sampling sites were purposively selected, the findings are exploratory and descriptive of the sampled herds only and should not be generalized as a nationally representative estimate for Pakistan.

### 2.4. Sample Size Determination and Blood Collection

The minimum sample size was calculated using the standard formula for estimating a population proportion, assuming 50% expected prevalence, a 95% confidence level, and 5% absolute precision, yielding a minimum sample size of 384 animals. This calculation assumes simple random sampling and a 50% expected prevalence, which gives the largest required sample size; no prior estimate was available for interface settings. Because animals were clustered within herds (mean of about 35 animals per herd), the effective sample size is smaller than the number of animals sampled (with a mean of about 35 animals per herd, an intracluster correlation of 0.05–0.30 would give design effects of about 2.7–11.1 and effective sample sizes of roughly 720–175 animals). The final sample size was increased to 1939 animals to allow descriptive subgroup comparisons by species, breed, geographic region, and other epidemiological variables.

A total of 1939 animals were sampled, including 1327 goats and 612 sheep. Approximately 5 mL of blood was collected aseptically from the jugular vein of each animal using sterile vacutainer tubes. Samples were transported on ice to the laboratory and centrifuged at 3000 rpm for 10 min to separate serum, which was stored at −20 °C until analysis.

### 2.5. Serological Testing

Detection of antibodies against PPRV was performed using a competitive enzyme-linked immunosorbent assay (c-ELISA) kit (ID Screen^®^ PPR Competition, IDVet, Montpellier, France) according to the manufacturer’s instructions. The assay uses a monoclonal antibody directed against the PPRV nucleoprotein and expresses results as percentage inhibition (PI). Samples with PI ≥ 50% were considered positive, whereas samples with PI < 50% were considered negative. Positive and negative controls supplied with the kit were included according to the manufacturer’s instructions. Each serum sample was tested once.

The diagnostic sensitivity and specificity of the ID Screen^®^ PPR Competition c-ELISA have been reported as 94.5% and 99.4%, respectively, based on validation against the virus neutralization test (VNT) [12]. The kit is validated for serum and plasma from sheep and goats, and the test principle and interpretation criteria are described in the WOAH Manual of Diagnostic Tests and Vaccines for Terrestrial Animals. The manufacturer reports a diagnostic sensitivity of >99% and specificity of >99% for the ID Screen PPR Competition based on internal validation data. Validation was not performed in the present study populations. Because the c-ELISA detects antibodies against PPRV regardless of their origin, seropositivity cannot distinguish between vaccine-induced and infection-induced antibodies. Therefore, vaccination history was recorded and considered during interpretation of the serological findings.

### 2.6. Data Collection

A structured questionnaire was developed and pre-tested/piloted on approximately 10% of the selected herds that were not included in the final study sample.

The final questionnaire captured the following information:

Animal-level variables: species (goat/sheep), age, sex, breed, body condition score, and reproductive status for females.

Herd-level variables: herd size, production system (extensive/semi-intensive), herd movement pattern (sedentary/nomadic/transhumant), introduction of new animals during the preceding 12 months, and contact with other herds at grazing or watering points.

Vaccination variables: vaccination status (vaccinated/unvaccinated), number of vaccine doses, date of most recent vaccination, and vaccination regularity (regular/irregular/none).

Livestock–wildlife interface variables: reported wildlife sightings near the herd, shared grazing areas with wild ruminants, shared water sources, and distance from the herd’s primary grazing area to the nearest protected-area boundary. Distance was measured as the straight-line distance from the coordinates of each herd’s primary grazing area, recorded using a handheld GPS device during field sampling, to the nearest boundary of the protected area, using Google Earth.Pro 7.3.6.9796. Distances were then assigned to the predefined categories (0–2, 3–5, 10–25, 25–30 and 30–40 km)

Questionnaires were administered in the local language by trained veterinary field staff. All responses were recorded using standardized data-collection forms and checked for completeness and consistency at the end of each sampling day.

### 2.7. Ethical Approval

The study was approved by the Department of Veterinary Microbiology, Faculty of Animal Husbandry and Veterinary Sciences, Sindh Agriculture University, Tandojam, Pakistan (approval code: MICR/153 of 2023; date of approval: 26 August 2023). All procedures involving animals were conducted in accordance with institutional guidelines for the care and use of animals in research.

### 2.8. Statistical Analysis

Data were entered and organized using Microsoft Excel and analyzed using IBM SPSS Statistics version 26. Descriptive statistics were used to calculate overall and stratified seroprevalence. Apparent seroprevalence was calculated as the number of c-ELISA-positive animals divided by the total number of animals tested, multiplied by 100.

Associations between seropositivity and categorical variables such as species, sex, breed, age group, vaccination status, and region were evaluated using chi-square (χ^2^) tests or Fisher’s exact test where expected cell frequencies were small.

Binary logistic regression analysis was performed to assess the association between potential factors and PPRV seropositivity. Crude odds ratios (ORs) and 95% confidence intervals (CIs) were estimated for individual explanatory variables, and site-adjusted odds ratios are reported where the data allowed.

Variables with *p* < 0.25 in the univariable analysis were considered for adjustment [13]. Because animals were sampled within herds and herds within sites, observations are not independent. A herd-level random-intercept model could not be fitted with the data available for this analysis; the reasons are stated in Section 4.3, and the alternative approaches used are described below. We therefore (i) estimated the odds ratio for goats versus sheep adjusted for site using logistic regression with site as a fixed effect, the Mantel–Haenszel estimator and a logistic mixed-effects model with a site random intercept, and tested heterogeneity of the species effect across sites (Woolf test); (ii) quantified between-site heterogeneity of prevalence (Cochran Q and I^2^) and a crude site-level intracluster correlation (ANOVA estimator); and (iii) performed a sensitivity analysis in which the variance of the crude estimates was inflated by the design effect, DEFF = 1 + (m − 1) × ICC, with mean cluster size m ≈ 35 and ICC values from 0.05 to 0.30, to identify the herd-level ICC above which each association would lose statistical significance. Distance, breed and site are largely confounded and were not modelled jointly.

Because sites and herds were purposively selected, confidence intervals and *p*-values describe sampling variability among the sampled herds only and should be interpreted as exploratory; they do not allow inference to the wider small-ruminant population.

## 3. Results

### 3.1. Sampling Distribution

A total of 1939 small ruminants were sampled from 56 herds across eight sites in four regions of Pakistan between January and June 2024. The study population comprised 1327 goats (68.4%) and 612 sheep (31.6%). The number of animals sampled per site ranged from 71 at Togh Mangara to 662 at Bahawalpur, with an overall mean of approximately 35 animals per herd (site-level range: 24–41 animals per herd). The sampling framework, including geographic coordinates, protected-area proximity, distance categories, number of herds, and animals sampled at each site, is presented in Table 1.

Coordinates represent the approximate centroid of the primary grazing area at each sampling site and are reported in decimal degrees (WGS 84). A herd was defined as a group of small ruminants managed together by a single household or herder. Herd numbers represent counts recorded in the field sampling register.

### 3.2. Overall Seroprevalence

The overall apparent seroprevalence of PPRV antibodies was 38.5% (747/1939; 95% CI: 36.4–40.7%). The observed seropositivity indicates substantial exposure to PPRV or prior PPRV vaccination among the sampled small-ruminant population. This interval treats animals as independent and understates uncertainty: assuming a herd-level intracluster correlation of 0.10 or 0.20 widens it to 34.0–43.0% and 32.5–44.5%, respectively, and prevalence varied markedly among sites (Cochran Q = 115.9, 7 df; I^2^ = 94%).

### 3.3. Species-Wise Seroprevalence

A marked difference in apparent seroprevalence was observed between species. Goats had a significantly higher seroprevalence (44.8%; 594/1327; 95% CI: 42.1–47.5%) than sheep (25.0%; 153/612; 95% CI: 21.7–28.6%). The association between species and seropositivity was statistically significant (χ^2^ = 69.07, *p*< 0.001), as shown in Table 2.

### 3.4. Site-Wise Seroprevalence

**Figure 2:**
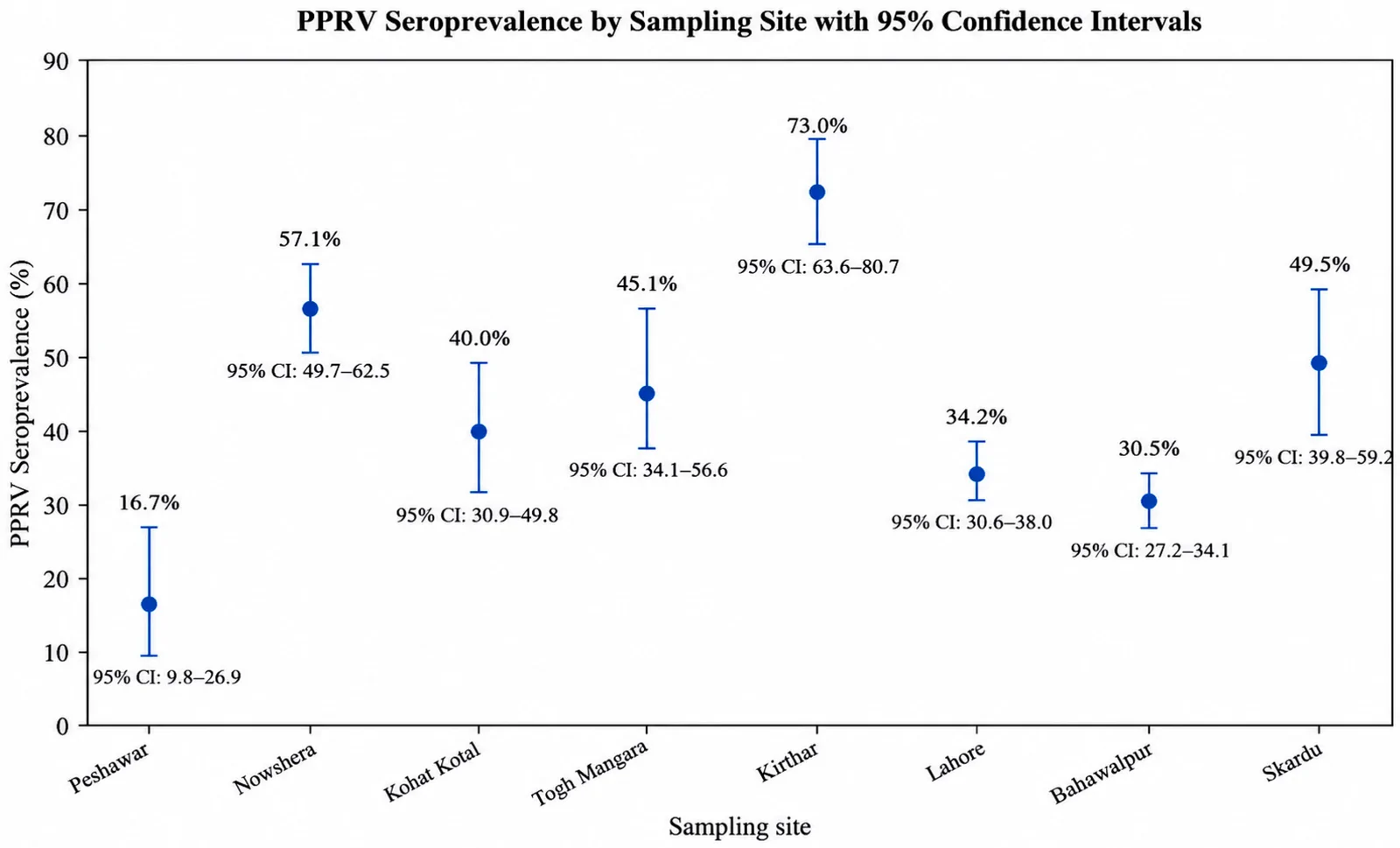
Site-wise seroprevalence of PPRV antibodies in small ruminants, with 95% confidence intervals.

Substantial site-level variation in apparent seroprevalence was observed among the eight sampling sites. Seroprevalence ranged from 16.7% at Peshawar to 73.0% at Kirthar National Park, as presented in Table 3 and Figure 2. The difference among sampling sites was statistically significant (χ^2^ = 127.65, 7 df, *p* < 0.001), indicating considerable geographic heterogeneity in PPRV antibody detection across the study areas.

### 3.5. Breed-Wise Seroprevalence

**Figure 3:**
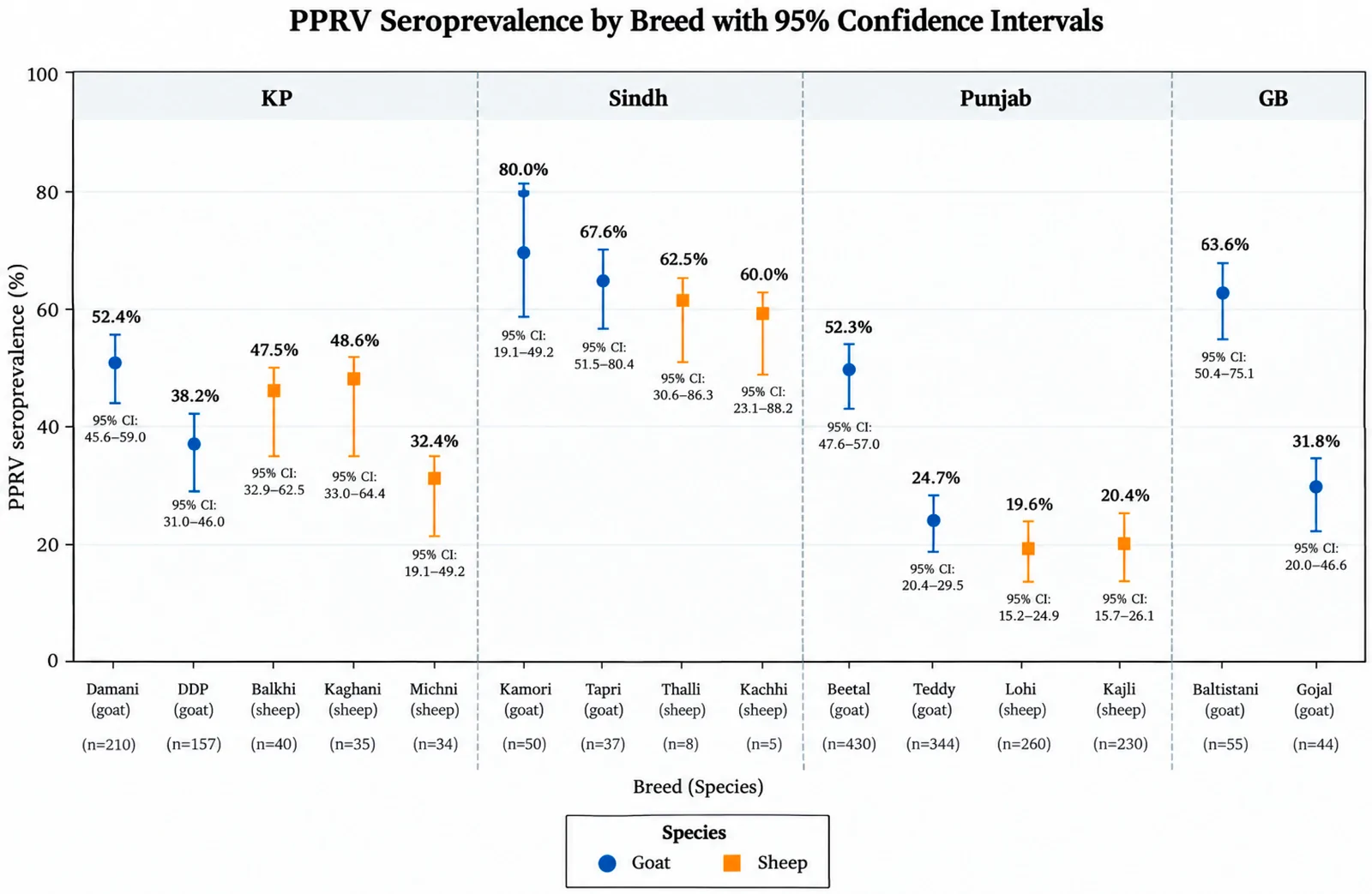
Breed-wise seroprevalence of PPRV antibodies, ordered by prevalence within species.

Apparent seroprevalence varied considerably among the 15 breeds represented in the study, as shown in Table 4 and Figure 3. Among goats, the highest seroprevalence was observed in Kamori (80.0%; 40/50) and Tapri (67.6%; 25/37), whereas the lowest was observed in Teddy (24.7%; 85/344) and Gojal (31.8%; 14/44). Among sheep, the highest seroprevalence was recorded in Thalli (62.5%; 5/8) and Kachhi (60.0%; 3/5), while the lowest was observed in Lohi (19.6%; 51/260) and Kajli (20.4%; 47/230).

Overall, seropositivity differed among breeds (χ^2^ = 221.66, 14 df, *p* < 0.001; four cells had expected counts below five, so this result should be interpreted with caution). Breed is strongly confounded with species and province, and breeds with fewer than 30 animals (Thalli, Kachhi) are not interpreted.

### 3.6. Sex-Wise Seroprevalence

Of the 1939 animals examined, 1039 (53.6%) were female and 900 (46.4%) were male. Apparent seroprevalence was higher among females (41.0%; 426/1039) than males (35.7%; 321/900). This difference was statistically significant (χ^2^ = 5.79, *p* = 0.016), as shown in Table 5.

### 3.7. Age-Wise Seroprevalence

Apparent seroprevalence varied across age categories, as shown in Table 6. Animals aged ≤ 1 year showed a seroprevalence of 34.0% (217/639), compared with 33.8% (270/800) among animals aged > 1–3 years and 52.0% (260/500) among animals aged > 3 years. In the univariable analysis, animals aged > 3 years had significantly higher odds of seropositivity than those aged > 1–3 years (crude OR = 2.13, 95% CI: 1.69–2.67, *p* < 0.001).

### 3.8. Herd Size and Management Characteristics

The 56 herds were categorized into three groups according to herd size. Twenty herds contained <50 animals, 20 contained 50–100 animals, and 16 contained >100 animals. These herds contributed 600, 700, and 639 sampled animals, respectively.

Apparent seroprevalence was 36.7% (220/600) among animals from herds with <50 animals, 35.7% (250/700) among those from herds containing 50–100 animals, and 43.3% (277/639) among those from herds containing >100 animals, as shown in Table 7. In the univariable analysis, animals from herds containing >100 animals had higher odds of seropositivity than those from herds containing 50–100 animals (crude OR = 1.38, 95% CI: 1.11–1.72, *p* = 0.004). Apparent seroprevalence was 36.7% (220/600) among animals from herds with <50 animals, 35.7% (250/700) among those from herds containing 50–100 animals, and 43.3% (277/639) among those from herds containing >100 animals, as shown in Table 7. In the univariable analysis, animals from herds containing >100 animals had higher odds of seropositivity than those from herds containing 50–100 animals (crude OR = 1.38, 95% CI: 1.11–1.72, *p* = 0.004). This association was sensitive to herd-level clustering and is reported as exploratory.

Additional herd-level characteristics recorded during the survey were production system, herd movement pattern, introduction of new animals during the preceding 12 months, and contact with other herds at grazing or watering points. These variables were recorded at herd level and are described in Section 2.6, but they were not analyzed against serological outcomes because, with only 56 herds and eight purposively selected sites, herd-level exposures cannot be separated from site effects without pseudo replication.

### 3.9. Vaccination Status

Vaccination history was considered when interpreting serological findings because the c-ELISA detects antibodies against PPRV irrespective of whether they result from vaccination or natural infection. Among the sampled animals, 639 were classified as regularly vaccinated, 500 as irregularly vaccinated, and 800 as unvaccinated.

Apparent seroprevalence was 37.1% (237/639) among regularly vaccinated animals, 32.4% (162/500) among irregularly vaccinated animals, and 43.5% (348/800) among unvaccinated animals, as shown in Table 8. In the univariable analysis, unvaccinated animals had higher odds of seropositivity than regularly vaccinated animals (crude OR = 1.31, 95% CI: 1.06–1.62, *p* = 0.014), whereas the association for irregularly vaccinated animals was not statistically significant (crude OR = 0.81, 95% CI: 0.64–1.04, *p* = 0.100). Because the c-ELISA cannot differentiate vaccine-induced antibodies from antibodies resulting from natural infection, these vaccination-associated findings should be interpreted cautiously. Vaccination categories were defined as follows: “regularly vaccinated” = animals with a documented history of at least one PPR vaccination in each of the preceding two years, with the most recent dose administered within 12 months before sampling; “irregularly vaccinated” = animals with a history of vaccination but with the most recent dose administered more than 12 months before sampling, or with an incomplete or interrupted schedule; “unvaccinated” = animals with no reported history of vaccination, no vaccination certificate, and no record of vaccination with the local veterinary officer. Vaccination status was recorded from owner recall and, where available, verified against vaccination certificates or records held by the local veterinary officer.

### 3.10. Livestock–Wildlife Interface and Distance from Protected Areas

Apparent seroprevalence varied according to the distance between sampling locations and the nearest protected-area boundary, as presented in Table 9. The highest seroprevalence was observed among animals sampled 3–5 km from a protected-area boundary (61.9%; 206/333), followed by those sampled 30–40 km away (49.5%; 49/99). Seroprevalence was 34.6% (84/243) in the 0–2 km category, 34.2% (206/602) in the 10–25 km category, and 30.5% (202/662) in the 25–30 km category. Because direct livestock–wildlife interactions were not measured, distance from the nearest protected-area boundary was used as a proxy for potential livestock–wildlife interface exposure. Accordingly, the observed distance-specific differences should not be interpreted as evidence of direct wildlife-mediated transmission. Distance categories are largely confounded with sampling site; therefore, these values are descriptive and do not allow inference on wildlife-related exposure.

### 3.11. Univariable Analysis of Factors Associated with PPRV Seropositivity

Univariable binary logistic regression was performed to evaluate associations between candidate explanatory variables and PPRV seropositivity. Species, breed, age group, sex, herd size, vaccination status, and distance from the nearest protected-area boundary were considered as candidate variables. Variables associated with seropositivity at *p* < 0.25 were considered for inclusion in the multivariable model.

Goat species, female sex, age > 3 years, herd size > 100 animals, unvaccinated status, and the 3–5 km and 30–40 km distance categories showed evidence of association with seropositivity, as shown in Table 10. Goats had more than twice the odds of seropositivity compared with sheep (crude OR = 2.43, 95% CI: 1.97–3.01, *p* < 0.001). Animals aged > 3 years had higher odds than those aged > 1–3 years (OR = 2.13, 95% CI: 1.69–2.67, *p* < 0.001). Animals from herds containing >100 animals also had higher odds compared with those from herds containing 50–100 animals (OR = 1.38, 95% CI: 1.11–1.72, *p* = 0.004).

The strongest association among the distance categories was observed for animals sampled 3–5 km from protected-area boundaries (OR = 3.12, 95% CI: 2.36–4.12, *p* < 0.001), while animals sampled 30–40 km away also showed higher odds compared with the 10–25 km reference category (OR = 1.88, 95% CI: 1.23–2.89, *p* = 0.004). Breed was also considered as a candidate variable because of the substantial variation in seroprevalence observed among breeds. These distance estimates are crude and confounded with site.

### 3.12. Adjustment for Site and Sensitivity to Herd Clustering

Because a herd-level clustered model was not possible with the data used here, we examined whether the main crude associations persisted after accounting for site and how sensitive they are to herd-level clustering (Table 11). After adjustment for site, goats remained more likely to be seropositive than sheep (OR = 2.19, 95% CI: 1.76–2.74, *p* < 0.001; Mantel–Haenszel OR = 2.18, 95% CI: 1.75–2.72; site random-intercept model OR = 2.20, 95% CI: 1.76–2.74). However, the species difference varied among the seven sites that sampled both species (OR from 0.83 at Kohat Kotal to 4.13 at Lahore; Woolf heterogeneity test, χ^2^ = 18.31, 6 df, *p* = 0.005), so the pooled odds ratio should be regarded as an average over heterogeneous site-specific effects.

Associations with age, herd size and vaccination status could not be adjusted for site with the available data. The sensitivity analysis shows that the association for age > 3 years (crude OR = 2.13) would remain significant unless the herd-level ICC exceeded about 0.30, whereas the associations for herd size > 100 animals (crude OR = 1.38) and for unvaccinated status (crude OR = 1.31) would lose significance at herd-level ICCs of only about 0.03 and 0.02, respectively. Intracluster correlations of this size or larger are common for infectious-disease serology; these two associations should therefore be regarded as exploratory and possibly attributable to clustering. A herd random-intercept model could not be fitted with the available data; the reasons are stated. The approaches used here, site-adjusted estimation, between-site heterogeneity assessment and sensitivity analysis using the design effect, are therefore presented as the best available alternatives, and the estimates should be interpreted as exploratory.

Because animals were sampled within herds and herds within sites, the estimates in Table 10 and Table 11 should be interpreted as exploratory. A model with a herd-level random intercept, which would give the herd-level ICC and cluster-adjusted odds ratios, was not possible with the data used here.

## 4. Discussion

This study provides one of the largest serological assessments of PPRV antibody seropositivity in small ruminants sampled in areas adjacent to protected wildlife habitats in Pakistan. A total of 1939 animals from 56 herds across eight sites in four regions were tested using a competitive ELISA. The overall apparent seroprevalence was 38.5%, with substantial variation across species, sites, breeds, age groups, herd sizes, and vaccination categories. These findings contribute to the growing body of evidence on PPRV epidemiology in Pakistan and provide region-specific data to inform vaccination and surveillance strategies aligned with the WOAH–FAO global eradication goal.

The overall apparent seroprevalence of 38.5% (95% CI: 36.4–40.7%) observed in this study is lower than the pooled estimate of approximately 51% reported in a recent systematic review and meta-analysis of Pakistani small ruminants [14]. However, our estimate falls within the wide range of regional estimates reported in that analysis, including approximately 61% in Sindh, 54% in Punjab, 44% in Khyber Pakhtunkhwa, and 51% in Gilgit-Baltistan. The overall estimate is also consistent with the 38.7% reported by [15] in the northern border region of Pakistan, and comparable to the 42.17% reported by [16] in southwestern Uganda.

The difference between our estimate and the meta-analytic pooled prevalence likely reflects several factors. First, our sampling frame spanned four agro-ecological zones with differing livestock densities, production systems, and vaccination coverage, leading to substantial within-study heterogeneity. Second, the c-ELISA used in this study detects antibodies against PPRV regardless of whether they arise from natural infection or vaccination, and our sample included both vaccinated and unvaccinated animals. The pooled estimate reported by [17] includes studies that vary widely in their vaccination status inclusion criteria, which limits direct comparison. Third, our estimate is derived from a purposively selected set of interface sites rather than a nationally representative sample; it should therefore be interpreted as representative of the sampled ecological settings, not of Pakistan as a whole. This limitation is consistent with the approach of [18], who also reported higher prevalence in Punjab (79.77%) among unvaccinated animals, and with [19], who reported 45.8% in southwestern Balochistan among animals without recent vaccination history.

Goats had significantly higher seroprevalence (44.8%) than sheep (25.0%) (χ^2^ = 69.07, *p* < 0.001; site-adjusted OR = 2.19, 95% CI: 1.76–2.74, *p* < 0.001; the species difference varied between sites, with ORs from 0.83 to 4.13). This species difference is consistent with findings from multiple settings. Ref. [17] reported 90.27% seroprevalence in goats compared with 68.75% in sheep in Punjab. Ref. [18] reported 50.4% in goats compared with 38.9% in sheep in Balochistan. Ref. [16] reported 46.27% in goats compared with 33.62% in sheep in Uganda. The consistency of this pattern across diverse ecological and management settings is compatible with higher exposure of goats to PPRV; possible, untested explanations include differences grazing behaviour, exploratory activity, herd management, and mixing with other animals.

Several biological and management factors may explain this differential exposure. Experimental infection studies have demonstrated that goats develop more severe clinical signs, higher viral loads, and prolonged shedding compared with sheep [19,20]. Behavioural differences, including goats’ greater curiosity, exploratory behaviour, and tendency to roam, may increase their contact with infected animals or contaminated environments. Additionally, husbandry practices in the study regions may involve goats being herded separately from sheep or allowed to browse more extensively, both of which may increase their exposure to contaminated environments or other animals. These explanations are hypotheses that were not tested in the present study. However, because the c-ELISA cannot distinguish infection-induced from vaccine-induced antibodies, these findings should be interpreted as evidence of differential exposure rather than differential biological susceptibility. These findings do not by themselves establish which species should be prioritized for vaccination; vaccination of both species remains important for PPR control.

Seroprevalence varied substantially across the eight sampling sites, ranging from 16.7% in Peshawar to 73.0% at Kirthar National Park (χ^2^ =127.65, 7 df, *p* < 0.001; Table 3). This heterogeneity is consistent with previous reports from Pakistan and neighbouring countries. Ref. [15] reported district-level seroprevalence ranging from 25.1% in Swat to 53.4% in Gilgit. Ref. [15] reported a range from 52.05% in Okara to 100% in Nankana. Ref. [18] reported a range from 40.7% in Mastung to 52.0% in Kech.

Several factors may explain the observed site-level variation. First, the sites differ in their proximity to protected wildlife areas, ranging from 0 to 2 km to 30–40 km. Second, they differ in production systems, livestock densities, and access to veterinary services. Third, they differ in vaccination coverage, which was not uniform across the study sites. Fourth, Kirthar National Park in Sindh has previously been identified as a high-prevalence region in the [17] meta-analysis, which reported a pooled Sindh prevalence of approximately 61%. Our site-level estimate of 73.0% at Kirthar is higher than that pooled estimate, possibly reflecting the specific interface setting sampled and the high goat density in the region.

The high seroprevalence at Kirthar National Park warrants particular attention. As discussed further below, proximity to wildlife habitat may increase opportunities for livestock–wildlife contact, though this study did not directly measure such contact. Similar wildlife-associated elevations in seroprevalence have been reported by [16] at the Lake Mburo wildlife interface in Uganda and by [21] at the livestock–wildlife interface in Punjab.

Seropositivity differed among breeds (χ^2^ = 221.66, 14 df, *p* < 0.001; descriptive, confounded with species and province), with marked variation across the 15 breeds sampled (Table 4). Among goats, Kamori (80.0%) and Tapri (67.6%) showed the highest seroprevalence, while Teddy (24.7%) and Gojal (31.8%) showed the lowest. Among sheep, Thalli (62.5%) and Kachhi (60.0%) showed the highest seroprevalence, while Lohi (19.6%) and Kajli (20.4%) showed the lowest.

This finding is consistent with the Punjab study by [17], which reported breed as the strongest single factor associated with PPRV seropositivity (χ^2^ = 200.89, *p* < 0.0001), with Makhi Cheeni (100%) and Beetal (97.72%) showing the highest and Lohi (20.45%) and Sindhi (30.76%) the lowest seroprevalence. The consistency of the breed effect across studies suggests that both intrinsic (genetic or immunological) and extrinsic (management, movement, and market exposure) factors may contribute to breed-specific patterns of exposure.

However, breed was not included in the adjusted analysis because it is strongly confounded with species and geography. Breed-level analyses in this study should therefore be interpreted as descriptive rather than as independent predictors of seropositivity. This is consistent with the approach of [16], who noted that breed effects in mixed-flock systems reflect relative epidemiological risk rather than direct biological susceptibility.

Females had higher seroprevalence (41.0%) than males (35.7%) (χ^2^ = 5.79, *p* = 0.016; crude OR = 1.25, 95% CI: 1.04–1.51). Sex could not be adjusted for site or for herd-level clustering with the available data, and it was not retained in the adjusted model; this association is therefore reported as exploratory.

The direction of the sex effect varies across published studies. Ref. [15] reported 42.9% in males compared with 37.6% in females, whereas Ref. [17] reported higher seroprevalence in males (85.77%) than in females (76.54%). Ref. [16] reported a similar direction to our study, with females showing higher prevalence in the univariable analysis but not retaining significance after adjustment. These divergent findings suggest that the sex effect is modest and likely confounded by species, age, and management practices. In our study, the lack of an adjusted analysis suggests that sex is not clearly associated with PPRV antibody status in this population.

Seroprevalence was similar between animals aged ≤1 year (34.0%) and > 1–3 years (33.8%), but substantially higher in animals aged > 3 years (52.0%) crude OR = 2.13, 95% CI: 1.69–2.67, *p* < 0.001. This association was robust to herd-level clustering in the sensitivity analysis. It would remain statistically significant unless the herd-level intracluster correlation exceeded approximately 0.30, which is above the range usually observed for infectious-disease serology. It is therefore reported as the most robust of the herd-level associations in this study, while acknowledging that a herd-level random-intercept model could not be fitted.

The higher seroprevalence in older animals is consistent with cumulative lifetime exposure to PPRV and has been reported across multiple settings. Ref. [16] reported higher prevalence ratios in animals aged > 1–3 years (aPR = 1.86) and > 3–5 years (aPR = 2.56) than in animals aged < 1 year. Ref. [18] reported higher seroprevalence in animals aged ≥ 1 year (AOR = 2.18). Ref. [15] reported that age was not statistically significant, though the direction of the association was similar.

The similar seroprevalence between ≤1 year and >1–3 years in our study may reflect maternally derived antibodies in young animals, which can persist for several months after birth and confer temporary protection. Similar patterns have been reported by [16], who noted that young animals may benefit from maternal immunity before becoming susceptible. This pattern is compatible with cumulative lifetime exposure and/or repeated vaccination and does not by itself indicate which age groups should be prioritized for vaccination.

Seroprevalence was highest in herds containing more than 100 animals (43.3%), compared with herds containing fewer than 50 animals (36.7%) and herds containing 50–100 animals (35.7%) crude OR = 1.38, 95% CI: 1.11–1.72, *p* = 0.004; this association is sensitive to herd-level clustering and is exploratory.

This finding is consistent with previous reports. Ref. [18] reported herd size ≥ 50 animals as the strongest independent risk factor for PPRV seropositivity in Balochistan (AOR = 3.12, 95% CI: 2.01–4.85). Ref. [15] reported that large herds (101–200 animals) had 1.7 times higher odds of positivity than small herds (OR = 1.79). Ref. [16] did not find herd size to be significant in their multivariable model, suggesting that the effect may vary with production system and sampling frame.

The association between larger herd size and higher seroprevalence is biologically plausible. Larger herds have higher animal density, greater contact rates between animals, more frequent introduction of new animals, and greater difficulty in implementing biosecurity measures and vaccination coverage. Larger herds are also more likely to be managed under extensive production systems with shared grazing lands, watering points, and market channels, all of which facilitate virus introduction and spread.

These findings suggest that larger herds may merit priority in surveillance and investigation; the data do not allow conclusions about the benefits of targeted vaccination or biosecurity measures.

Regularly vaccinated animals had seroprevalence of 37.1%, irregularly vaccinated animals 32.4%, and unvaccinated animals 43.5% (crude OR for unvaccinated vs. regularly vaccinated = 1.31, 95% CI: 1.06–1.62, *p* = 0.014; Table 8). Irregularly vaccinated animals had lower odds of seropositivity than regularly vaccinated animals, though this finding was not statistically significant (OR = 0.81, 95% CI: 0.64–1.04, *p* = 0.100).

These findings must be interpreted with caution because the c-ELISA cannot distinguish between vaccine-induced and infection-induced antibodies. The observed seroprevalence in vaccinated animals therefore reflects either prior natural exposure or vaccine-induced immunity, so seropositivity in vaccinated animals cannot be attributed to either cause. This limitation is acknowledged by [17], who addressed it by sampling only unvaccinated animals, and by [16], who reported true prevalence using the Rogan–Gladen estimator. In our study, we sampled both vaccinated and unvaccinated animals, which is both a strength (allowing us to assess the association between vaccination status and seropositivity) and a limitation (preventing us from attributing seropositivity exclusively to natural infection).

The vaccination results are descriptive and do not allow conclusions about vaccine effectiveness. Seropositivity in unvaccinated animals is more likely to reflect past natural exposure, although owner-reported vaccination status may be misclassified. An analysis restricted to the 800 unvaccinated animals, in which antibody positivity is more interpretable as evidence of past natural exposure, showed associations of similar direction to the full cohort for species and age, with wider confidence intervals owing to the smaller sample. This subgroup analysis cannot demonstrate active infection and does not remove the possibility of misclassification of vaccination status.

### 4.1. Livestock–Wildlife Interface

Seroprevalence varied with distance from the nearest protected-area boundary, with the highest seroprevalence (61.9%) observed in the 3–5 km category, followed by the 30–40 km category (49.5%), the 0–2 km category (34.6%), the 10–25 km category (34.2%), and the 25–30 km category (30.5%) (crude OR for 3–5 km vs. 10–25 km = 3.12, 95% CI: 2.36–4.12, *p* < 0.001; Table 9).

The observed pattern does not show a clear monotonic relationship between proximity to protected areas and seroprevalence. The highest seroprevalence in the 3–5 km category likely reflects the specific sites included in that group (Nowshera and Kirthar National Park), both of which had high seroprevalence for reasons that may be unrelated to wildlife proximity. The 30–40 km category contains only Skardu, which had a seroprevalence of 49.5% and may reflect different ecological and management conditions. Distance categories are largely confounded with site, so an independent effect of distance cannot be estimated from these data.

These findings should not be interpreted as evidence of direct wildlife-mediated transmission, because wildlife were not sampled and direct livestock–wildlife contact was not measured. Instead, proximity to protected areas was used as a proxy for potential interface exposure. This approach has precedent in the literature. Ref. [17] reported that farms reporting wildlife sightings on the farm had 43% higher prevalence ratios than farms that did not (aPR = 1.43, 95% CI: 1.07–1.91, *p* = 0.015). Ref. [22] reported elevated seroprevalence in livestock sampled at the livestock–wildlife interface in Punjab. Ref. [15] reported elevated seroprevalence in regions with large wild animal populations, particularly in Gilgit and Chitral.

The findings do, however, suggest that further investigation of livestock–wildlife contact pathways is warranted, particularly at sites such as Kirthar National Park where seroprevalence was highest and where wild ungulates are present. Future studies should combine livestock and wildlife serology, direct behavioural observation of contact, and molecular characterization of circulating PPRV strains to establish whether cross-species transmission is occurring. This is consistent with the One Health approach advocated by WOAH and FAO for PPR eradication.

### 4.2. Methodological Strengths

This study has several methodological strengths. It is one of the largest PPRV serosurveys conducted in Pakistan to date, with 1939 animals from 56 herds across three provinces and one administrative territory. It spans eight sites representing four agro-ecological zones and five distance categories from protected areas. It includes breed-level, sex-level, age-level, herd-size-level, and vaccination-status-level analyses, providing a comprehensive epidemiological profile of PPRV exposure in the sampled populations. It uses a validated c-ELISA kit (ID Screen^®^ PPR Competition) with reported diagnostic sensitivity of 94.5% and specificity of 99.4%. It reports both apparent seroprevalence and statistical associations using logistic regression.

### 4.3. Limitations

This study has several limitations that should be acknowledged. First, the cross-sectional design permits identification of associations but not causal inference. The observed relationships between associated factors and seropositivity may reflect underlying confounders that were not measured. Second, the c-ELISA detects antibodies against PPRV but cannot distinguish vaccine-induced from infection-induced seropositivity. Seropositivity represents evidence of PPRV antibody presence resulting from previous infection and/or vaccination, rather than active infection. Future studies should use DIVA-compatible diagnostic tests where available.

Third, wildlife was not sampled and direct livestock–wildlife contact was not measured. The livestock–wildlife interface was inferred from proximity to protected areas rather than from direct observation, which limits the strength of any conclusions about wildlife-mediated transmission.

Fourth, the purposive selection of study sites means that findings are representative of the sampled ecological and interface settings rather than of Pakistan as a whole. The study does not provide a nationally representative estimate.

Fifth, herd-level clustering could not be modelled: animals were sampled within herds and herds within sites, so standard errors, confidence intervals and *p*-values for the crude estimates are too small, particularly for herd-level factors such as herd size and vaccination status. We addressed this only by adjusting the species effect for site and by a sensitivity analysis based on assumed intracluster correlations, which showed that the herd-size and vaccination associations would not survive modest herd-level clustering. Only eight purposively selected sites were available, so site effects could not be separated from other site-level characteristics. Future studies should use multilevel models with herd as a random effect and a larger number of sites.

Sixth, age and vaccination status were correlated in the sampled population and their separate effects could not be fully disentangled. The group sizes coincided numerically (639, 800 and 500 in both classifications), and because a herd-level random-intercept model could not be fitted, no formal collinearity diagnostics (cross-tabulation and variance inflation factors) could be produced. We therefore report the age- and vaccination-related findings as exploratory, and we do not present age and vaccination as independent predictors.

Seventh, the analysis relied on owner recall for age and vaccination history, which may be subject to recall bias. Where vaccination certificates were available, these were used to verify the reported information, but not all owners could provide documentation.

Eighth, the absence of clinical and virological data limits the ability to distinguish between past exposure and active infection. Seropositivity alone cannot establish whether animals were currently infected or had recovered from prior exposure.

Ninth, each serum was tested once, so samples near the cut-off may have been misclassified; sampling covered only January–June, so seasonal variation in herd movement, kidding/lambing and vaccination could not be assessed; and the c-ELISA was not validated in the study populations. Distance categories were confounded with site and herd-level analyses were underpowered.

### 4.4. Implications for PPR Control in Pakistan

The findings of this study have several implications for PPR control in Pakistan.

First, the high overall seroprevalence indicates substantial PPRV antibody prevalence in the sampled regions; virological confirmation of current circulation was not available. Strengthened vaccination campaigns are needed, particularly in high-prevalence sites such as Kirthar National Park.

Second, the higher seroprevalence in goats compared with sheep indicates that goats may merit particular attention in surveillance, while vaccination of both species remains important.

Third, the higher seroprevalence in animals older than three years may reflect cumulative exposure and/or repeated vaccination and does not by itself indicate which age groups should be prioritized.

Fourth, the higher seroprevalence in herds with more than 100 animals suggests that larger herds may merit priority in surveillance and investigation.

Fifth, vaccination remains a cornerstone of PPR control, but the present data cannot be used to evaluate vaccine effectiveness.

Sixth, the observed variation across sites with differing proximity to protected areas suggests that the livestock–wildlife interface warrants further investigation as part of a One Health approach to PPR control. This is consistent with the WOAH–FAO global eradication strategy, which recognizes the need to integrate livestock and wildlife surveillance.

## 5. Conclusions

This exploratory, purposively sampled survey found that antibodies against PPRV were common in small ruminants sampled near the selected protected wildlife areas (apparent seroprevalence 38.5%), with marked differences among sites. Goats, animals older than three years and animals from herds with more than 100 animals had higher crude odds of seropositivity; the goat–sheep difference persisted after adjustment for site (OR = 2.19, 95% CI: 1.76–2.74) but varied between sites, whereas the herd-size association is sensitive to herd-level clustering. Because vaccinated animals were included and wildlife were not sampled, the results indicate neither natural-infection prevalence, vaccine effectiveness nor transmission at the livestock–wildlife interface. They do identify sites and animal groups that merit intensified surveillance, and they support future studies that combine livestock and wildlife serology, direct measurement of contact and virus sequencing to inform One Health control of PPR toward the 2030 eradication goal.

## Figures and Tables

**Table 1:** Sampling framework: province, district, sampling site, coordinates, protected area, distance to boundary, herds, and animals sampled.

Province	District/Area	Sampling Site	Latitude (°N)	Longitude (°E)	Nearest Protected Area	Distance (km)	Herds (*n*)	Sheep (*n*)	Goats (*n*)	Animals (*n*)
Khyber Pakhtunkhwa	Peshawar	Palosi/Peshawar Zoo area	34.02513	71.48677	Peshawar Zoo	0–2	3	10	62	72
Khyber Pakhtunkhwa	Nowshera	Nizampura	33.75910	72.03190	Manglot National Park	3–5	8	50	183	233
Khyber Pakhtunkhwa	Kohat	Kohat Kotal	33.58656	71.45064	Kotal Wildlife Park	0–2	4	23	77	100
Khyber Pakhtunkhwa	Kohat	Togh Mangara	33.56540	71.55410	Togh Mangara Safari Park/Game Reserve	0–2	3	26	45	71
Sindh	Jamshoro	Around Kirthar National Park	25.65000	67.53330	Kirthar National Park	3–5	4	13	87	100
Punjab	Lahore	Lahore	31.38110	74.21200	Lahore Safari/Wildlife Park	10–25	15	225	377	602
Punjab	Bahawalpur	Bahawalpur	29.33330	72.01670	Lal Suhanra National Park	25–30	16	265	397	662
Gilgit-Baltistan	Skardu	Skardu	34.98140	75.46010	Deosai National Park	30–40	3	0	99	99
Total							56	612	1327	1939

**Table 2:** Species-wise seroprevalence of PPRV antibodies in sheep and goats.

Species	Tested (*n*)	Positive (*n*)	Seroprevalence (%)	95% CI
Goat	1327	594	44.8	42.1–47.5
Sheep	612	153	25.0	21.7–28.6
**Total**	1939	747	38.5	36.4–40.7

**Table 3:** Site-wise seroprevalence of PPRV antibodies in sheep and goats across the eight sampling sites.

Province	Site	Sheep Tested	Goat Tested	Total Tested	Sheep Positive	Goat Positive	Total Positive	Prevalence (%)	95% CI
KP	Peshawar	10	62	72	1	11	12	16.7	9.8–26.9
KP	Nowshera	50	183	233	24	109	133	57.1	50.7–63.3
KP	Kohat Kotal	23	77	100	10	30	40	40.0	30.9–49.8
KP	Togh Mangara	26	45	71	12	20	32	45.1	34.1–56.6
Sindh	Kirthar	13	87	100	8	65	73	73.0	63.6–80.7
Punjab	Lahore	225	377	602	37	169	206	34.2	30.6–38.0
Punjab	Bahawalpur	265	397	662	61	141	202	30.5	27.2–34.1
GB	Skardu	0	99	99	0	49	49	49.5	39.8–59.2
Total		612	1327	1939	153	594	747	38.5	36.4–40.7

Note: KP = Khyber Pakhtunkhwa; GB = Gilgit-Baltistan; CI = confidence interval.

**Table 4:** Breed-wise seroprevalence of PPRV antibodies among sheep and goats.

Province	Species	Breed	Tested (*n*)	Positive (*n*)	Prevalence (%)	95% CI
KP	Goat	Damani	210	110	52.4	45.6–59.0
KP	Goat	DDP	157	60	38.2	31.0–46.0
KP	Sheep	Balkhi	40	19	47.5	32.9–62.5
KP	Sheep	Kaghani	35	17	48.6	33.0–64.4
KP	Sheep	Michni	34	11	32.4	19.1–49.2
Sindh	Goat	Kamori	50	40	80.0	19.1–49.2
Sindh	Goat	Tapri	37	25	67.6	51.5–80.4
Sindh	Sheep	Thalli	8	5	62.5	30.6–86.3
Sindh	Sheep	Kachhi	5	3	60.0	23.1–88.2
Punjab	Goat	Beetal	430	225	52.3	47.6–57.0
Punjab	Goat	Teddy	344	85	24.7	20.4–29.5
Punjab	Sheep	Lohi	260	51	19.6	15.2–24.9
Punjab	Sheep	Kajli	230	47	20.4	15.7–26.1
GB	Goat	Baltistani	55	35	63.6	50.4–75.1
GB	Goat	Gojal	44	14	31.8	20.0–46.6

Note: KP = Khyber Pakhtunkhwa; GB = Gilgit-Baltistan; DDP = Dera Din Panah; CI = confidence interval.

**Table 5:** Sex-wise seroprevalence of PPRV antibodies.

Sex	Tested (*n*)	Positive (*n*)	Seroprevalence (%)
Female	1039	426	41.0
Male	900	321	35.7
Total	1939	747	38.5

**Table 6:** Age-wise seroprevalence of PPRV antibodies.

Age Category	Tested (*n*)	Positive (*n*)	Seroprevalence (%)
≤1 year	639	217	34.0
>1–3 years	800	270	33.8
>3 years	500	260	52.0
Total	1939	747	38.5

**Table 7:** Herd-size-wise seroprevalence of PPRV antibodies.

Herd Size	Herds (*n*)	Animals (*n*)	Positive (*n*)	Seroprevalence (%)
<50 animals	20	600	220	36.7
50–100 animals	20	700	250	35.7
>100 animals	16	639	277	43.3
Total	56	1939	747	38.5

**Table 8:** Seroprevalence according to vaccination status.

Vaccination Status	Tested (*n*)	Positive (*n*)	Seroprevalence (%)
Regularly vaccinated	639	237	37.1
Irregularly vaccinated	500	162	32.4
Unvaccinated	800	348	43.5
Total	1939	747	38.5

**Table 9:** Seroprevalence by distance to the nearest protected-area boundary.

Distance (km)	Sites Included	Herds (*n*)	Animals (*n*)	Positive (*n*)	Seroprevalence (%)	95% CI
0–2	Peshawar, Kohat Kotal, Togh Mangara	10	243	84	34.6	28.9–40.8
3–5	Nowshera, Kirthar	12	333	206	61.9	55.9–66.4
10–25	Lahore	15	602	206	34.2	30.6–38.0
25–30	Bahawalpur	16	662	202	30.5	27.2–34.1
30–40	Skardu	3	99	49	49.5	39.8–59.2
Total		56	1939	747	38.5	36.4–40.7

Note: CI = confidence interval.

**Table 10:** Univariable (crude) logistic regression analysis of factors associated with PPRV seropositivity.

Variable	Comparison	Positive/Tested (*n/N*)	Apparent Seropositivity (%)	Crude OR	95% CI	*p*-Value	Overall *p*-Value (χ^2^, df)
Species	Sheep (reference)	153/612	25.0	1.00	Reference	—	<0.001 (69.07, 1)
	Goat vs. sheep	594/1327	44.8	2.43	1.97–3.01	<0.001	
Sex	Male (reference)	321/900	35.7	1.00	Reference	—	0.016 (5.79, 1)
	Female vs. male	426/1039	41.0	1.25	1.04–1.51	0.016	
Age	>1–3 years (reference)	270/800	33.8	1.00	Reference	—	<0.001 (51.66, 2)
	≤1 year vs. >1–3 years	217/639	34.0	1.01	0.81–1.26	0.934	
	>3 years vs. >1–3 years	260/500	52.0	2.13	1.69–2.67	<0.001	
Herd size	50–100 animals (reference)	250/700	35.7	1.00	Reference	—	0.009 (9.49, 2)
	<50 animals vs. 50–100	220/600	36.7	1.04	0.83–1.31	0.722	
	>100 animals vs. 50–100	277/639	43.3	1.38	1.11–1.72	0.004	
Vaccination status	Regular (reference)	237/639	37.1	1.00	Reference	—	<0.001 (16.84, 2)
	Irregular vs. regular	162/500	32.4	0.81	0.64–1.04	0.100	
	Unvaccinated vs. regular	348/800	43.5	1.31	1.06–1.62	0.014	
Distance	10–25 km (reference)	206/602	34.2	1.00	Reference	—	<0.001 (105.87, 4)
	0–2 km vs. 10–25 km	84/243	34.6	1.02	0.74–1.39	0.923	
	3–5 km vs. 10–25 km	206/333	61.9	3.12	2.36–4.12	<0.001	
	25–30 km vs. 10–25 km	202/662	30.5	0.84	0.67–1.07	0.160	
	30–40 km vs. 10–25 km	49/99	49.5	1.88	1.23–2.89	0.004	

Note: OR = odds ratio; CI = 95% confidence interval; *n/N* = positive/tested animals. Crude odds ratios were obtained from univariable logistic regression, with the following reference categories: sheep for species, male for sex, >1–3 years for age, 50–100 animals for herd size, regular vaccination for vaccination status, and 10–25 km for distance. *p*-values for individual comparisons were based on the Wald test, whereas overall *p*-values were obtained using Pearson’s χ^2^ test. Animals were not adjusted for herd-level clustering in the crude analysis; site-adjusted estimates and a sensitivity analysis for herd-level clustering are presented in Table 11. Distance categories are largely confounded with sampling site and are shown for description only.

**Table 11:** Species effect adjusted for site, and sensitivity of crude associations to herd-level clustering.

Analysis	Estimate (95% CI)	Comment
Goat vs. sheep, crude	OR 2.43 (1.97–3.01)	1327 goats, 612 sheep
Goat vs. sheep, site fixed effect	OR 2.19 (1.76–2.74)	7 sites with both species
Goat vs. sheep, Mantel–Haenszel	OR 2.18 (1.75–2.72)	Stratified by site
Goat vs. sheep, site random intercept	OR 2.20 (1.76–2.74)	Mixed-effects logistic model
Heterogeneity of species effect across sites	Woolf χ^2^ = 18.31, 6 df, *p* = 0.005	Site ORs 0.83 to 4.13
Between-site heterogeneity of prevalence	Cochran Q = 115.9, 7 df; I^2^ = 94%	Crude site ICC about 0.08
Age > 3 vs. >1–3 years	Crude OR 2.13 (1.69–2.67)	Loses significance only if herd ICC > about 0.30
Herd size > 100 vs. 50–100 animals	Crude OR 1.38 (1.11–1.72)	Loses significance if herd ICC > about 0.03
Unvaccinated vs. regular vaccination	Crude OR 1.31 (1.06–1.62)	Loses significance if herd ICC > about 0.02
Overall prevalence 38.5%, 95% CI if herd ICC = 0.10/0.20/0.30	34.0–43.0/32.5–44.5/31.3–45.7	Naive CI 36.4–40.7; DEFF 4.4/7.7/11.1

Note: OR = odds ratio; CI = 95% confidence interval; ICC = intracluster correlation coefficient; DEFF = design effect. Site-adjusted estimates use the seven sites with both species (Skardu had no sheep). Threshold ICCs were obtained by inflating the variance of the crude odds ratio by DEFF = 1 + (m − 1) × ICC (m = mean animals per herd) until the 95% CI included 1; they assume the exposure is constant within herds and are therefore conservative for age and sex, which vary within herds.

## Data Availability

The original contributions presented in this study are included in the article. Further inquiries can be directed to the corresponding authors.

## Abbreviations

The following abbreviations are used in this manuscript:

CI	Confidence Interval
c-ELISA	competitive enzyme-linked immunosorbent assay
FAO	Food and Agriculture Organization
IBM	International Business Machines
mL	Millilitre
OR	Odds ratio
PPR	Peste des Petits Ruminants
SPSS	Statistical Packages for social sciences Statistical Package for the Social Sciences
WOAH	World Organisation for Animal Health

## References

[1] Kabir A., Kalhoro D.H., Abro S.H., Kalhoro M.S., Yousafzai H.A., Shams S., Khan I.U., Lochi G.M., Mazari M.Q., Baloch M.W. (2019). Peste des petits ruminants: A review. Pure Appl. Biol..

[2] Munir M. (2014). Role of wild small ruminants in the epidemiology of peste des petits ruminants. Transbound. Emerg. Dis..

[3] FAO (2021). Peste des Petits Ruminants: Global Disease Eradication Strategy 2020–2030.

[4] Kardjadj M. (2018). Epidemiological situation of transboundary animal diseases in North African countries—Proposition of a regional control strategy. Trop. Anim. Health Prod..

[5] George J. (2017). Economic Impact of Contagious Caprine Pleuropneumonia and Peste des Petits Ruminants in Pastoral Communities of Ngorongoro and Coastal Districts, Tanzania. Ph.D. Thesis.

[6] de Haan N.C., Kimani T., Rushton J., Lubroth J., Munir M. (2014). Why is small ruminant health important—Peste des petits ruminants and its impact on poverty and economics?. Peste des Petits Ruminants Virus.

[7] Maluleke W., Mphatheni M.R., Nkosi S.L. (2022). A systematic study on stock theft contributory factors during the South African lockdown. Int. J. Res. Bus. Soc. Sci..

[8] Meena M., Swapnil P., Barupal T., Sharma K. (2019). A review on infectious pathogens and mode of transmission. J. Plant Pathol. Microbiol..

[9] Pruvot M., Fine A.E., Hollinger C., Strindberg S., Damdinjav B., Buuveibaatar B., Chimeddorj B., Bayandonoi G., Khishgee B., Sandag B. (2020). Outbreak of peste des petits ruminants among critically endangered Mongolian saiga and other wild ungulates, Mongolia, 2016–2017. Emerg. Infect. Dis..

[10] Ayebazibwe C., Akwongo C.J., Waiswa J., Ssemakula O., Akandinda A., Barasa M., Nkamwesiga J., Lule P., Mabirizi A., Lule P. (2022). Peste des petits ruminants (PPR) in Uganda: Assessment of animal health systems and coordination mechanisms. Front. Vet. Sci..

[11] Kock R.A., Osofsky S.A. (2005). What is this infamous “wildlife/livestock disease interface?” A review of current knowledge for the African continent. Conservation and Development Interventions at the Wildlife/Livestock Interface: Implications for Wildlife, Livestock and Human Health.

[12] Libeau G., Prehaud C., Lancelot R., Colas F., Guerre L., Bishop D.H.L., Diallo A. (1995). Development of a competitive ELISA for detecting antibodies to the peste des petits ruminants virus using a recombinant nucleobrotein. Res. Vet. Sci..

[13] Hosmer D.W., Lemeshow S., Sturdivant R.X. (2013). Applied Logistic Regression.

[14] Amin Z.S., Shafee M., Asadullah, Iqbal Z., Ahmad S., Raza M., Abbassi B.H.A., Mastoi A.H. (2026). Seroprevalence and Risk Factor Analysis of Peste des Petits Ruminants (PPR) in Small Ruminants of South-Western Balochistan, Pakistan. Pak. Vet. J..

[15] Munibullah, Li Y., Munib K., Zhang Z., Zhang Z. (2024). Prevalence and associated risk factors of peste des petits ruminants in selected districts of the northern border region of Pakistan. BMC Vet. Res..

[16] Musiime E., Bwambale K., Babirye P., Lumu P., Mutebi F., Mugizi D.R., Onyuth H. (2026). Seroprevalence of Peste des Petits Ruminants (PPR) and associated risk factors in selected sub-counties of Lyantonde district, South Western Uganda. BMC Vet. Res..

[17] Kabir A., Kamboh A.A., Abubakar M., Rahman A.U., Jabbar M.T., Shafiq M., Lin M. (2025). Peste Des Petits Ruminants in Pakistan: Current Status, Challenges and Prospects for Vaccine Development. Vaccines.

[18] Alvi M.A., Yan H.B., Kharl R.B., Ali A., Zeeshan M., Jia W.Z., Ren S., Meng X., Zhu X., Ghafoor M. (2026). Seroprevalence and associated risk factors of Peste des Petits Ruminants virus in small ruminants of Punjab, Pakistan. Transbound. Emerg. Dis..

[19] Rehman A., Memon M.I., Lakho A.A., Usman M., Munir A., Solangi N.A., Jan S.U., Amir F.A., Khan M., Kabir A. (2025). Seroprevalence and risk factors of peste des petits ruminants in goats from Khipro, District Sanghar, Sindh. Res. J. Vet. Pract..

[20] Kumar P., Tripathi B.N., Sharma A.K., Kumar R., Sreenivasa B.P., Singh R.P., Dhar P., Bandyopadhyay S.K. (2004). Pathological and immunohistochemical study of experimental peste des petits ruminants virus infection in goats. J. Vet. Med. Ser. B.

[21] Enchery F., Hamers C., Kwiatek O., Gaillardet D., Montange C., Brunel H., Goutebroze S., Philippe-Reversat C., Libeau G., Hudelet P. (2019). Development of a PPRV challenge model in goats and its use to assess the efficacy of a PPR vaccine. Vaccine.

[22] Rahman A.U., Abubakar M., Rasool M.H., Manzoor S., Saqalein M., Rizwan M., Munir M., Ali Q., Wensman J.J. (2016). Evaluation of risk factors for peste des petits ruminants virus in sheep and goats at the wildlife-livestock interface in Punjab province, Pakistan. BioMed Res. Int..

